# Supervising and Controlling Unmanned Systems: A Multi-Phase Study with Subject Matter Experts

**DOI:** 10.3389/fpsyg.2016.00568

**Published:** 2016-05-24

**Authors:** Talya Porat, Tal Oron-Gilad, Michal Rottem-Hovev, Jacob Silbiger

**Affiliations:** ^1^Department of Primary Care & Public Health Sciences, King's College LondonLondon, UK; ^2^Department of Industrial Engineering and Management, Ben Gurion University of the NegevBeer Sheva, Israel; ^3^HFE Independent ConsultantTel-Aviv, Israel; ^4^Synergy Integration Ltd.Tel-Aviv, Israel

**Keywords:** unmanned aerial systems, control ratio, UAV, decision support systems, DSS, automation, macrocognition, human factors

## Abstract

Proliferation in the use of Unmanned Aerial Systems (UASs) in civil and military operations has presented a multitude of human factors challenges; from how to bridge the gap between demand and availability of trained operators, to how to organize and present data in meaningful ways. Utilizing the Design Research Methodology (DRM), a series of closely related studies with subject matter experts (SMEs) demonstrate how the focus of research gradually shifted from “how many systems can a single operator control” to “how to distribute missions among operators and systems in an efficient way”. The first set of studies aimed to explore the modal number, i.e., how many systems can a single operator supervise and control. It was found that an experienced operator can supervise up to 15 UASs efficiently using moderate levels of automation, and control (mission and payload management) up to three systems. Once this limit was reached, a single operator's performance was compared to a team controlling the same number of systems. In general, teams led to better performances. Hence, shifting design efforts toward developing tools that support teamwork environments of multiple operators with multiple UASs (MOMU). In MOMU settings, when the tasks are similar or when areas of interest overlap, one operator seems to have an advantage over a team who needs to collaborate and coordinate. However, in all other cases, a team was advantageous over a single operator. Other findings and implications, as well as future directions for research are discussed.

## Introduction

The continuing proliferation in the use of UASs in both civil and military operations has presented a multitude of human factors challenges, including assessing the cognitive capabilities of one operator to simultaneously supervise and control multiple platforms, evaluating the advantages and disadvantages of an individual operator vs. a team, and finding meaningful ways to organize and present data. Underlying many of these challenges is the issue of how automation capabilities can best be utilized to assist human operators in handling increasing complexity and workload (Fern et al., 2011).

When the first unmanned aerial systems (UASs) were introduced in the 1980s, engineers and military leaders were content with their ability to extend capabilities of intelligence perception beyond the capacities that were there before. Once these technological advancements became part of a routine, it became evident that the ratio of personnel vs. crafts issue will rise. There are multiple reasons why managers and leaders are interested in reducing the man-machine control ratio, only to mention a few: fewer operators mean less need for training, less diversity in training, and reduced costs of manpower and training.

The focus on operator-UAS ratio corroborated even more in light of the US Office of the Secretary Defense Roadmap for unmanned aircraft systems (UASs: 2005-2030)^1^, which delineates the need to investigate the “appropriate conditions and requirements under which a single pilot would be allowed to control multiple airborne UA [unmanned aircraft] simultaneously.” Since then, till today the question of how many UASs or UAVs (Unmanned Aerial Vehicles) can one operator control or supervise has become a vital question that many researchers try to answer (e.g., Chen et al., 2013; Goodrich and Cummings, 2014).

Cummings et al. (2007a) proposed a hierarchical control model to portray control loops for a single operator in control of one UAV or multiple systems. In this three-level model, the innermost loop (Flight controls) represents the need for basic guidance and motion control (i.e., keeping the aircraft in stable flight) and is the most critical. If operators must interact in this loop, the cost will be very high since this loop requires significant cognitive resources. The second loop (Navigation) represents the actions that should be executed to meet mission constraints, such as routes to waypoints, time on targets, and avoidance of threat areas. The outermost loop (Mission and payload management) represents the highest levels of control—decisions which require knowledge-based reasoning that must be made to meet overall mission requirements. Health and status monitoring are tasks that cross all three loops, where the operator is required to perform continuous supervision to ensure that all systems are operating within normal limits. Hence, in order for one operator to be able to control multiple systems, operators will need to interact primarily at the outermost loop via a mission and payload manager while relegating routine navigation and motion control tasks to the automation. For example, given such significant autonomy, one operator could control 4–5 vehicles (Cummings et al., 2007a) and apply supervisory control for up to 12 vehicles (Cummings and Guerlain, 2007).

Higher levels of automation will enable operators to increase the number of unmanned systems they control and supervise, however, extensive use of automation can also introduce human performance costs such as loss of situation awareness, skill degradation, complacency, increased mental workload (Parasuraman et al., 2000) and automation bias (Mosier and Skitka, 1996). Hence, supervisory control of multiple UASs raises questions concerning how to balance system autonomy and human interaction (Calhoun et al., 2011, 2013). Furthermore, the challenge of incorporating automation in one vehicle is replaced by the need to keep the human “in the loop” of the activities for all vehicles (Ruff et al., 2002). Careful system design can mitigate performance costs and can be achieved by: allowing flexibility in the design of function allocation (i.e., which tasks will be performed by the human and which will be performed by the system), the level of automation to be implemented within each function (Parasuraman et al., 2000; Chen et al., 2013; Gu et al., 2014), and the operators' level of trust in the automation (Clare et al., 2015). Eventually, when flight control becomes fully automated, operators will manipulate the payloads rather than fly the vehicles (e.g., Cooper and Goodrich, 2008).

Ruff et al. (2002) compared the effects of automation level and decision-aid fidelity on the number of simulated remotely operated vehicles (ROVs) that could be successfully controlled by a single operator during a target acquisition task. Their results indicated that an automation level incorporating management-by-consent had clear performance advantages over the more autonomous (management-by-exception) and less autonomous (manual control) levels of automation. Calhoun et al. (2011) used a UAV simulation environment to evaluate two applications of autonomy levels across two primary control tasks: allocation (assignment of sensor tasks to vehicles) and router (determining vehicles' flight plans). Their results showed that performance on both primary tasks and many secondary tasks was better when the level of automation was the same across the two sequential primary tasks. Thus, having the level of automation similar across closely coupled tasks reduced mode awareness problems, which can negate the intended benefits of a fine-grained application of automation.

Adaptive automation (AA) alters the level of automation dynamically during operation. This allows the automation to account for individual differences and allows the automation to be more flexible, context-dependent, and user-specific (Saqer et al., 2011). Wilson and Russell (2007) demonstrated that the customization of automation and difficulty level to the individual operator had greater potential benefit than AA developed based on group performance means. Cummings et al. (2010) examined the impact of increasing automation re-planning rates on operator performance and workload when supervising a decentralized network of heterogeneous unmanned vehicles. They claimed that the future of one operator controlling multiple UVs requires automated planners, which are faster than humans at path planning and resource allocation. They examined three increasing levels of re-planning, and showed that rapid re-planning can cause high operator workload, ultimately resulting in poorer overall system performance. Calhoun et al. (2013) designed an interface enabling pilots to flexibly change the role of automation during the mission, transitioning between four control modes ranging from manual to high level “plays.” Their results showed that this approach is promising for single operator supervisory control of multiple UASs, however participants claimed that flexibility should be increased even more, enabling the operator to employ multiple control modes in a single task.

While automation can definitely increase the number of UASs a single operator can supervise and control, Hancock et al. (2007) raised a concern with the ongoing debate over how many UASs should or can a single operator control. The functional design questions that were raised were: (a) should researchers and designers continue to strive for a higher ratio, and, (b) if they decide to go forward in this direction, what is the modal number? As with all design questions, the immediate answer was simple: It depends. To be sure, the human being as the ultimate adaptive system may be able to demonstrate multiple UAS control, but we consider this an instance of what design can do, not what design should do. In response, John Senders commented that “*with appropriate control and display systems, the handling of more than one machine remains both useful and practical. Simultaneous (actually, appropriately sampled) control of many high-order systems by one operator was demonstrated to be feasible when the displays of attitude are appropriately quickened. Henry P. Birmingham demonstrated this many decades ago by showing excellent simultaneous control of 2 two-dimensional, third-order systems (Birmingham and Taylor, 1954*^2^) …. *Even modestly intelligent design would allow multiple UAVs and multiple displays to be searched or monitored efficiently with good connectivity between the displays. The individual operator is therefore the appropriate unit of analysis only when such bottlenecks occur at that level. More generally, if one views the collective team as an integrated, flexible system, then the very question of the UAV:Operator ratio itself becomes irrelevant.”*

After decades of field practice, the importance of operational use of UASs in combat and in civil operations has increased tremendously. Different team configurations consisting of Multiple Operators and Multiple UASs (MOMU) are nowadays evaluated (e.g., Mekdeci and Cummings, 2009; Gao et al., 2014), implying that indeed the operator to UAS ratio has become an outcome but not a target of its own.

MOMU is a relatively new operational setup for covering areas of interest, particularly in reconnaissance missions. It is highly relevant for homeland security and surveillance operations. A mode of one operator controlling multiple UASs can often increase the cognitive burden of its operators. MOMU setups aim to prevent high operator workload and low situation awareness, and can be very advantageous in offloading tasks to distribute workload among operators. Furthermore, MOMU setups can be advantageous also in terms of utilization of assets, as they contribute to increasing payload efficiency and system effectiveness. However, MOMU settings initiate new challenges for operators as they require switching of information sources, i.e., tasks, missions, video feeds, or camera manipulations and responsibilities among operators.

Switching is a time-critical, cognitively demanding task. Cognitive costs of switching may be loss of orientation and situation awareness (SA), increase in workload, and decrease in efficiency of verbal team communication and coordination. Consequently, switching between sources can disrupt operator performance (Draper et al., 2008; Squire and Parasuraman, 2010), and generate slower and less accurate responses compared to performing a single type of task (Allport et al., 1994; Monsell, 2003). In MOMU environments, where operators need to handoff aircrafts, payloads, targets, or missions to each other, switches may have a vital effect on mission accomplishment.

Over the past decades our team has advanced and improved operational concepts for UASs operators in surveillance and recon missions. Like most others, our studies began with examining the UAS to operator ratio, then to how to increase capacity of a single operator by utilizing tools and automation modes, which gradually shifted toward the MOMU framework. Here we report and revisit these multi-phase studies. Our goal is to demonstrate how the focus of research and practice moved toward a more collaborative operational concept that enables distribution of work and assets among multiple operators. We demonstrate the progress that has been occurring in this human-unmanned system research and how we perceive it should be further directed. We begin with operator to UAS ratio studies. Then, we demonstrate how the MOMU concept evolved. Lastly, we discuss why the changes in UASs control concepts are relevant for other less mature human-robot control domains.

The series of studies has been utilizing the Design Research Methodology (DRM; Blessing and Chakrabarti, 2009). DRM is sometimes called “Improvement Research” emphasizing the problem solving/performance-improving nature of the activity. It enables researchers and analysts to rapidly develop and test prospective improvements, deploy what they have learned about what works, and add to their knowledge to continuously improve the performance of the system (Vaishnavi and Kuechler, 2004). Our aim was to look at the problem from different levels of activity (e.g., supervise, control, mission management), settings (individual vs. team), resources (number of operators, number of vehicles), and automation levels.

In this paper, we do not portray details of every single step in each individual study. Our focus was on the design implications that stemmed from each study phase. This was a conscious strategy, not to be reductionists *per se*, but to allow examination of the operational concept issues from a higher perspective. All the evaluations that are presented were conducted with highly experienced UAS operators (subject matter experts; SMEs) which is necessary for DRM.

## Methods

**Figure 1:**
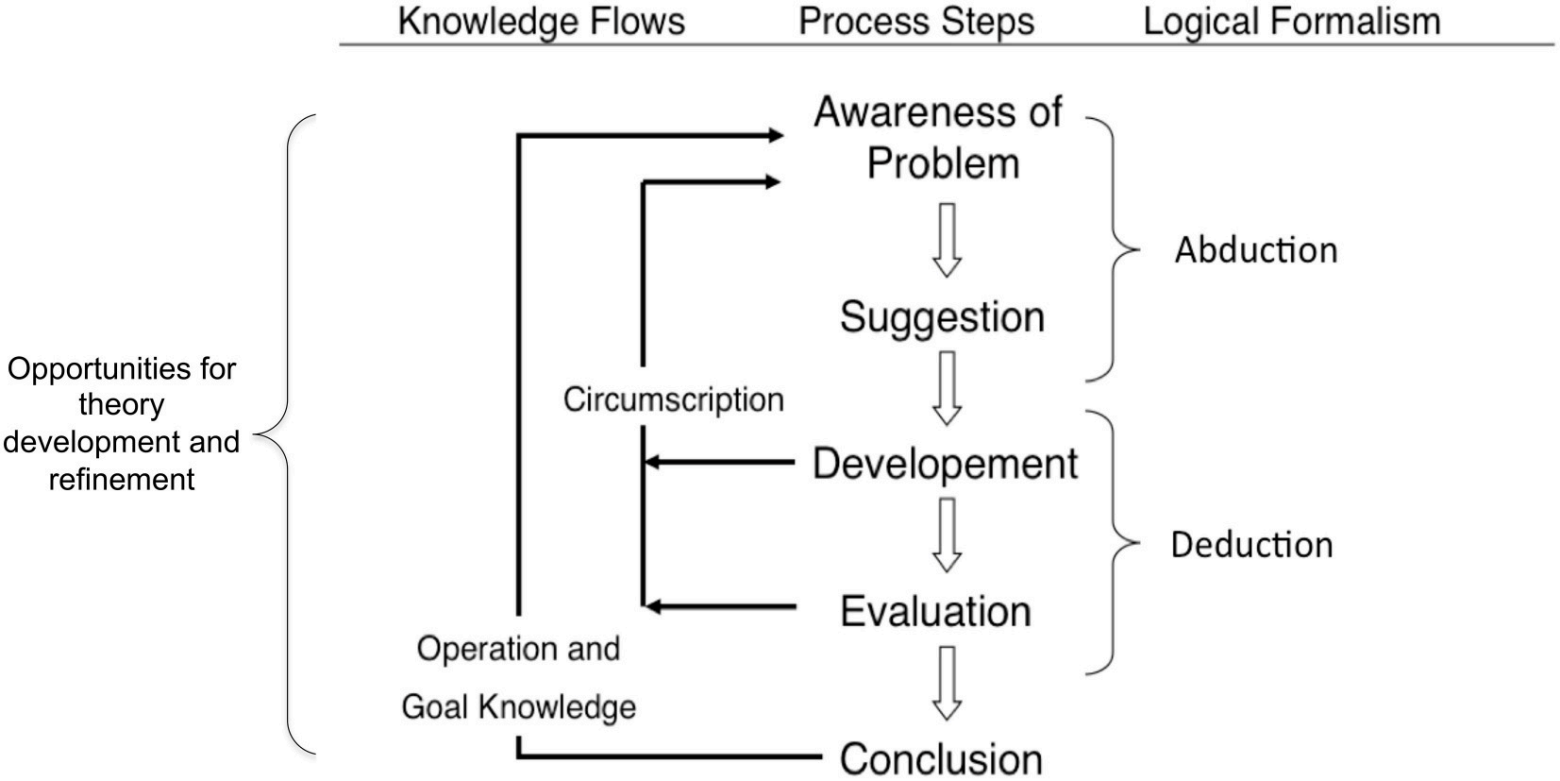
**Reasoning in the Design Research Cycle (cf. Kuechler and Vaishnavi, 2008)**.

We utilized the DRM, with SMEs which focuses on what works, for whom, and under what conditions. In this model (see Figure 1) all designs begin with *Awareness of a problem*; then usually from the existing knowledge of the problem area, solutions are *suggested*, after the suggestion phase, there is an attempt to implement an artifact according to the suggested solution—the *Development* phase. Partially or fully successful implementations are then *evaluated* with potential users. *Development, Evaluation* and further *Suggestions* are frequently iteratively performed in the course of the research (design) effort. The basis of the iteration, the flow from partial completion of the cycle back to *Awareness of the Problem*, is indicated by the *Circumscription* arrow. *Conclusion* indicates termination of a specific design project. New knowledge production is indicated by the arrows labeled *Circumscription* and *Operation and Goal Knowledge* (Vaishnavi and Kuechler, 2004; Kuechler and Vaishnavi, 2008). The goal of DRM is to help design research become effective and efficient by making the most out of valuable resources and applying gathered knowledge “on the move.” It is particularly suitable for complex interactive systems.

**Figure 2:**
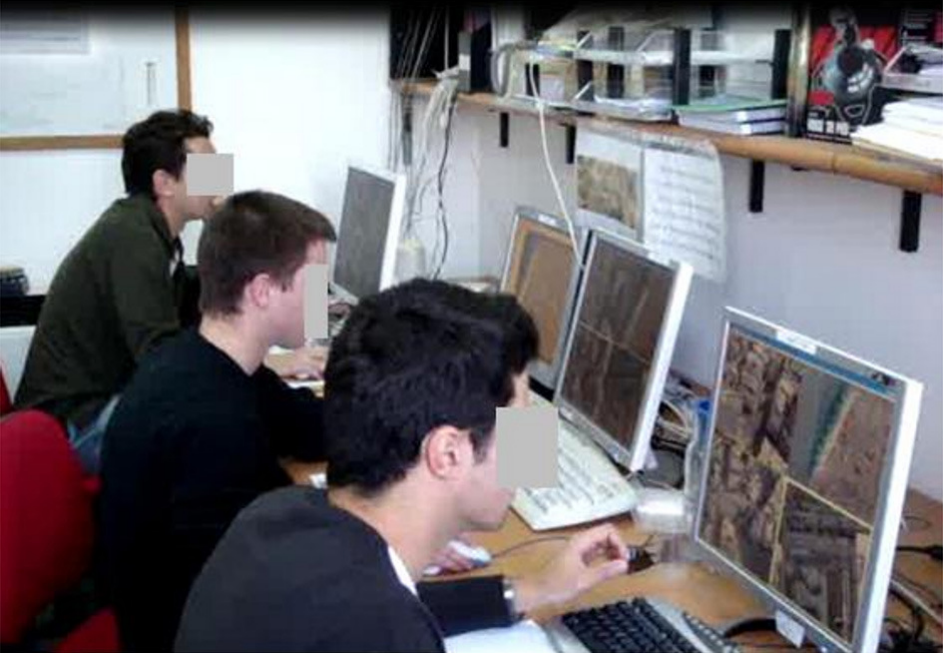
**The simulated environment**. In the configuration shown here three operators are collaboratively operating three UASs at the same time.

The studies took place at a designated laboratory at Synergy Integration Ltd. which was set up to resemble a typical UAS control room (see Figure 2). The work environment was simulated, but “true to life,” mimicking UAS military operators' work, who need to operate UASs while placed in a remote designated cabin. The lab consisted of several connected workstations containing a simulation system, which could be configured according to the task and needs (i.e., number of vehicles, individual vs. team operation, time limitations, use of decision support tools, etc.). In this setting, cognitive tasks such as planning, detecting problems, and managing uncertainty (macro-cognitive processes) could be evaluated. Level of automation and mission components were chosen using arrangements similar to the control loops of Cummings et al. (2007a).

## Studies

In the following we describe four studies, with their sub-conditions. The earlier two studies examined the operator/platform ratio in several operational scenarios and tasks. The first study examined the number of UASs one operator can supervise (health and status monitoring). The second study examined the number of UASs one operator can control (Mission and payload management) at a single instance. Studies 3 and 4 compared performance of one operator vs. a team of operators controlling the same number of UVs (MOMU studies). Study 3 took place in the UAS environment, while Study 4 took place in the UGV (unmanned ground vehicle) environment. This enabled us to further examine commonalities between the domains of operation. In the following, each study with its different experimental conditions is described.

### Study 1

**Figure 3:**
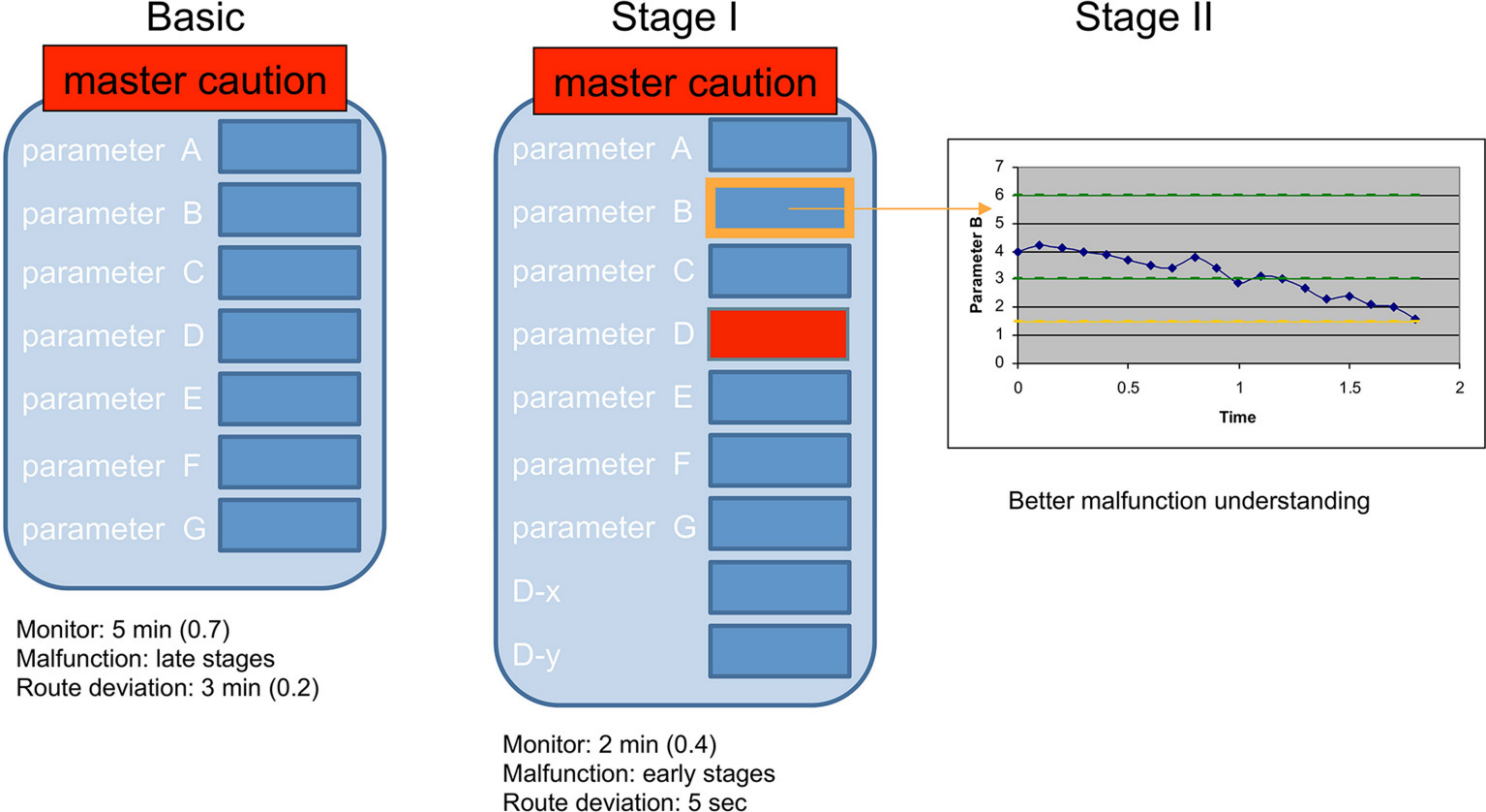
**Study 1 illustration**. **Left:** the original health data form; **Mid:** The modified health data form with two-step (orange and red) fault indicators (condition B); **Right:** Graphic presentation of a trend in the zoom-in view of one health parameter (condition C).

*Problem:* Starting the project, in what may now seem archaic for the UAS domain, health monitoring was identified as the main attention pitfall for operators. Back then, operators had to check the system's health repeatedly while they were performing the flight mission. Displayed health data had to be compared manually against a manufacturer checklist, an error prone process with heavy reliance on memory and specifically prospective memory (see Figure 3).

The first study aimed to facilitate the health monitoring task, using automation and tools in order to increase the efficiency and the number of UAV's that one operator could supervise simultaneously.

*Study question:* How many UAVs can one operator supervise (health monitoring) efficiently?

*Participants:* Five highly experienced male operators. All are reserve soldiers in active duty. They had 4–7 years of experience in operating military UASs (mean: 5.2), and their age ranged from 23 to 30 (mean: 26.6). SMEs were compensated for their time. The same five participants performed each one of the study conditions, hence a within-subject design was used. Since in DRM one makes incremental design changes, and this process takes time, there was a significant time gap between the different conditions (at least 1 month).

#### Initial state—manual, sequential supervising

##### Task

1:5—one operator manually supervised five UAVs of the same kind (utilizing a paper-based checklist).

##### Procedure

For each UAV, 13 health indices were displayed numerically on a form. In addition, two location indices were displayed on a map (X-Y coordinates, related to the pre-defined route). To evaluate the health status of the UAV, participants had to compare the values on the on-line form to a paper-based checklist with the appropriate value ranges. On the screen, the operator could view the health data of only one UAV at a time (i.e., the task required sequential browsing of the health forms). Operators performed continuous manual health monitoring by comparing each index in each form to the desired values written in the hard-copy. While doing this, operators had to relate to different flight stages, as health values varied as a function of flight stage.

##### Results

The cycle time to supervise one UAV was very long—5 min (*SD* = 0.7). The time to detect a fault depended on its location on the form and most faults were detected in late stages of the flaw. Detecting the fault source was almost impossible and took on average 13 min (*SD* = 6). Deviations from the planned route were detected late, after an average of 3 min (*SD* = 0.2), hence, only after there was a meaningful deviation from the route on the map (scale: 1:50,000).

Operators indicated that the task was difficult and exhaustive within less than 1 h of supervising. They complained on high workload and that they could not imagine succeeding in supervising another (6th) UAV.

#### Condition A—simultaneous supervising

##### Task

1:5—one operator manually supervised five UAVs with two changes relative to the initial state condition.

##### Suggestion—design change from initial state

To facilitate manual health monitoring, two design implementations were introduced: (1) for each data item an intact indication was added, depending on the flight stage: Intact, Warning (5% lower or higher than the intact value), or Fault; (2) all UAV health data forms were displayed simultaneously.

##### Results

The cycle time to supervise one UAV has decreased from 5 to 2 min (*SD* = 0.4). Most faults were detected in early stages (an average of 5 s to detect a fault). Detection of fault source and route deviations did not improve or differ from the initial state.

#### Condition A+—like a but with more systems

##### Task

1:10—one operator supervised manually 10 UAVs with the same design as in Condition A.

##### Suggestion—design change from condition A

Five additional UAVs were added to the supervising task. The limitation to 10 was due to screen size (which enabled displaying up to 10 UAV health forms simultaneously).

##### Results

Similar results to condition A—the cycle time to supervise one UAV remained 2 min on average (*SD* = 0.4). Most faults were detected in early stages (average of 5 s to detect a fault). Detection of fault source and route deviations did not improve from the initial state.

#### Condition B—grouping the health indices

##### Task

1:10–1:20—Operators started with supervising 10 UAVs. During the evaluation, UAVs were added gradually until a single operator was supervising 20 UAVs at a time. To facilitate supervising, the 12 health indices were grouped into four categories.

##### Suggestion—design change from condition A+

There was a change in the display design: the two location indices and one health index were removed (the focus was now only on health parameters). The remaining 12 health indices were grouped into four meaningful categories (e.g., engine, communication, etc.). For each of them, three intact indications were displayed: Intact, Warning and Fault. The shape of the indication icon implied on the contained data in each category. For example, the group containing communication measures (increase/decrease) had an indication icon of arrows pointing up or down.

For each UAV only group indications were displayed on the health data form. The operator could open the full form by clicking on the indication group.

##### Results

Results were similar to the ones in condition A. Operators reported upon high workload and a feeling of losing control once the 17th UAV was added.

#### Condition B+—single indicator for each system

##### Task

1:10–1:20—the operator started supervising 10 UAVs. During the study UAVs were added gradually until stopped at one operator supervising 20 UAVs with a change in the way intact indications were displayed.

##### Suggestion—design change from condition B

The four group indications used in Condition B for each UAV were replaced with one intact indication (icon) for each UAV placed on the command and control map. The operator could click on the icon and view the detailed form. In addition, an alert was added for location deviation.

##### Results

Results were similar as in condition A, except for the time to detect deviations from route which was dramatically shortened to 5 s on average (instead of 3 min in previous conditions). Operators succeeded in supervising 15-17 UAVs.

#### Condition C—addition of malfunction/health problem trends

##### Task

1:10–1:20—Operators started with supervising 10 UAVs. During the study UAVs were added gradually until stopped at one operator supervising 20 UAVs. The major change was the addition of a graph display to identify trends in health measures.

##### Suggestion—design change from condition B+

For each indicator, a graph displaying its measured values and intact indications was added. The graph was displayed once the user clicked on the measure value from the health data form. The purpose of this condition was to evaluate if time based information on any specific indication could decrease the time it took for operators to detect the fault source (i.e., aimed to facilitate better malfunction source detection, see Figure 3).

##### Results

Results were the same as in condition A, except for the major improvement in the time to detect the fault source, which decreased to less than 5 min in 95% of the cases (instead of an average of 13 min in all previous conditions). The ability to view the behavior of the health-related measure over time has helped the operators to understand and detect the source of the fault. The downside of this measure is that it is only suitable for mature systems where the number of faults is relative small, and there is a clear well established link between the health-related measure and its source. Operators succeeded in supervising up to 10 UAVs, mainly because here, more attention was allocated to detecting the source of the fault than previously, and there was not enough time for all the faults to be further examined.

#### Study 1 summary

After performing the first study with its three main conditions, it is possible to claim that one experienced operator can supervise up to 15 UAVs efficiently using the level of automation, the indication tools and the task characteristics described in conditions B and B+. Nevertheless, since health monitoring is only part of mission demands, it was necessary to further investigate the issue of mission and payload management control in Study 2.

### Study 2

*Problem:* The “classical” ratio concern; there was a requirement to increase the number of UAVs that one operator can control.

*Study question:* How many UAVs can one operator control (mission and payload management) efficiently and how can this ratio be improved.

*Participants:* Ten highly experienced male operators (SMEs) with similar military background and skills. They had 3–10 years of experience (mean: 5.6) —7 SMEs in operating military UASs and 3 SMEs in operating other types of military electro-optical sensors. Their age ranged from 23 to 30 (mean: 26). SMEs were compensated for their time.

#### Condition A—one to one vs. one to two

##### Task

1:1 vs. 1:2—One operator tracks a moving target with one UAV vs. two UAVs.

**Figure 4:**
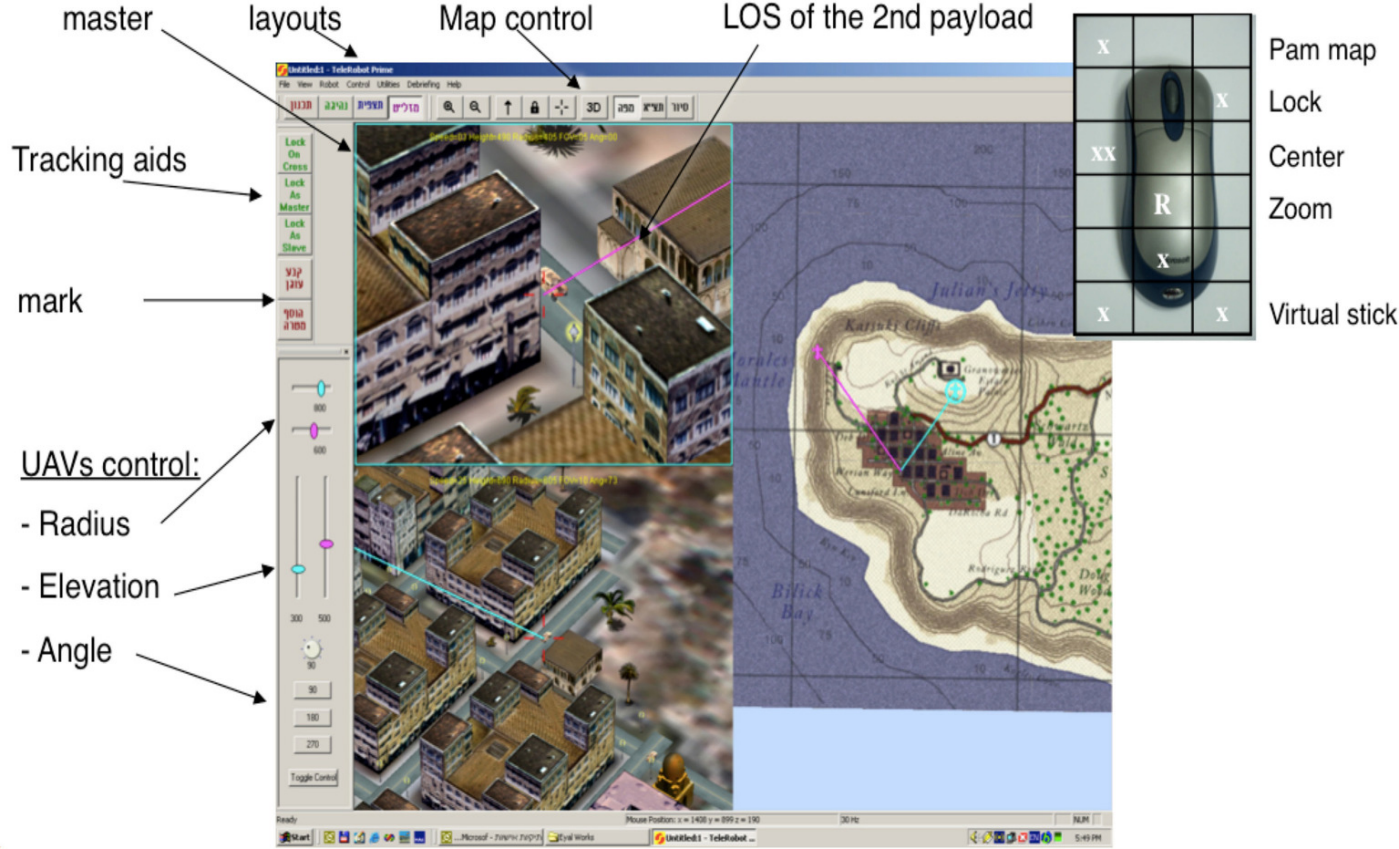
**Twin UAV operation screen configuration and operational device (mouse)**.

Comparing performance of tracking a moving target with two UAVs (Twin UAV setup) vs. a single UAV, in an urban environment. Twin UAV is a “pair of UAVs” handled and operated as one system by one operator (see Figure 4). Either UAV can serve as the master while the other one is slaved and vice versa. Hence, only one payload needs to be controlled at a time, and the enslaved UAV positions itself relative to the master. The UAVs control is at a high level of automation via payload management. Various parameters need to be set by the operator for each UAV, prior to each sortie and can be changed during the sortie (altitude, turn radius, camera field-of-view and position shift angle between the UAVs).

##### Procedure

The experiment consisted of six experimental scenarios. Each scenario was performed twice, once with one UAV and once with the Twin UAV configuration. The order was counterbalanced among participants. Each trial began with the target vehicle in a specified position. The vehicle then started moving and the operator was asked to keep it in sight as continuously as possible (a lock-on Target feature could be used when the target was visible). Task difficulty depended on the number of similar vehicles in the scene (varied from 5 to 9) and on obstructions when buildings occluded the target. The target vehicle looked similar to other vehicles but had a unique mark. The four easier scenarios lasted 3 min each and the two more difficult ones lasted 4 min. Instructions about the user interface and the task, a demonstration, and four Twin UAV and one single UAV training trials preceded the experimental phase.

##### Results

**Figure 5:**
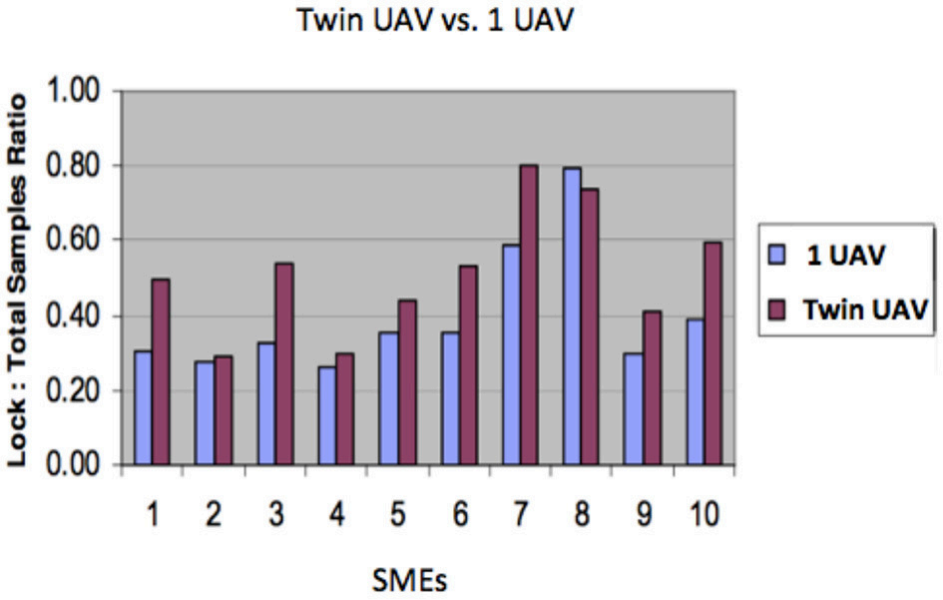
**Comparison of lock-on time (i.e., the proportion of time during which the target was visible and locked by at least one UAV) with “Twin UAV” setup and with a single UAV, by participant**.

Sampling ratio (time spent in “Lock-on target” mode relative to the total duration of the scenario) was significantly (*p* < 0.05) higher when participants used the Twin UAV (average 0.42, SD 0.12) than the single UAV (average 0.31, SD 0.04). No significant interaction was found between scenario and UAV setup (twin vs. single). Figure 5 shows the results for each participant.

#### Condition B —one to three

##### Task

**Figure 6:**
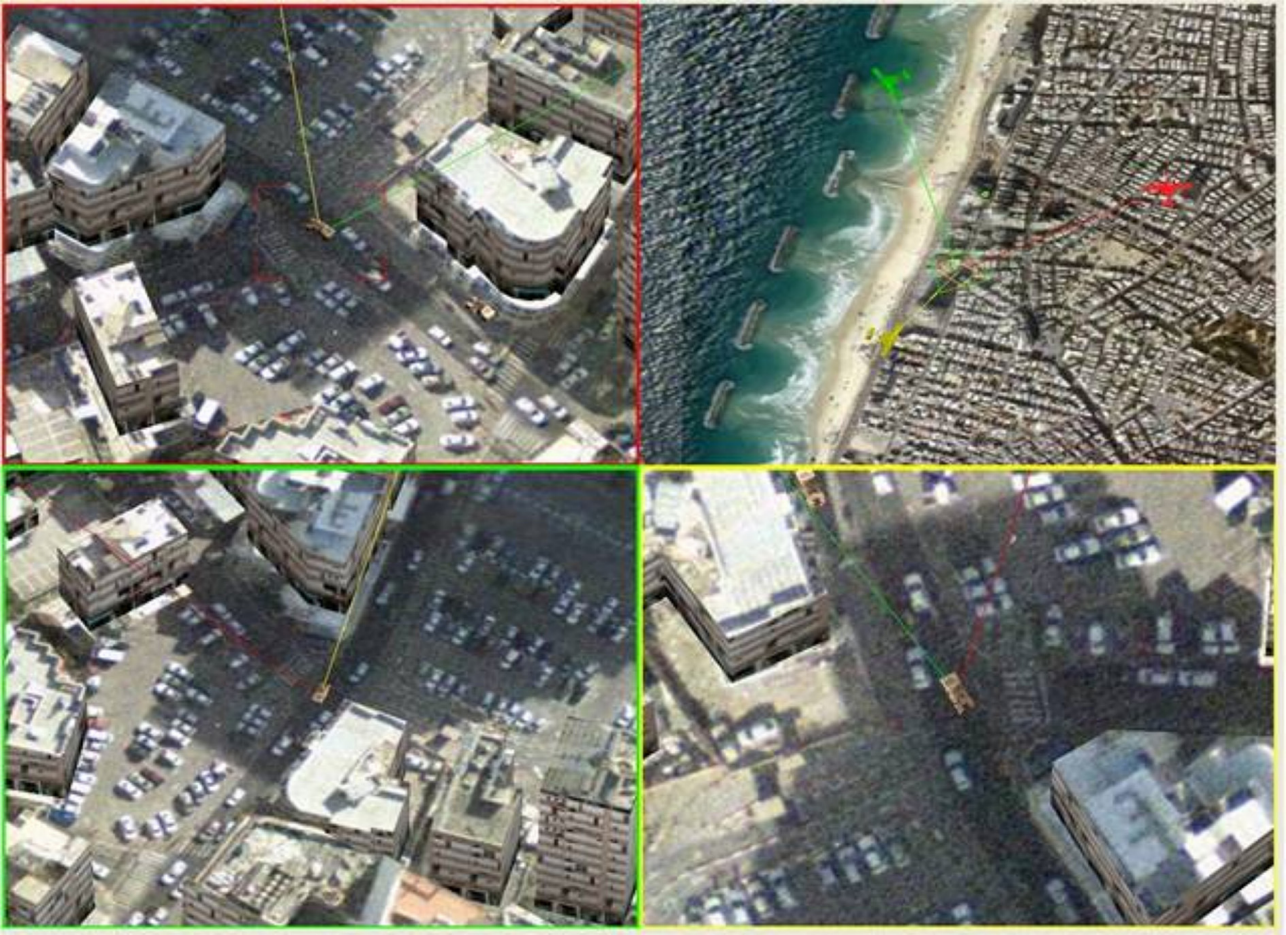
**Tri-UAV display, the screen is divided into four areas: video feeds of the three UAVs marked with a colored frame for identification (upper left, lower left, and lower right windows), and a command and control map (upper right window)**. Note that all three UAVs are shown on the map.

1:3—Here a more complex operational mission was used; one operator was required to guard a building, track a suspicious vehicle, and scan the shoreline using three UAVs (Tri-UAV), see Figure 6.

##### Procedure

The Tri-UAV display contained video feed windows for each payload and a common map. The operator controlled the display using a mouse and a keyboard. The mouse enabled the operator to move the cursor between the map window and the video feed windows, and point to a specific location.

The task took place in a densely built urban environment. The operator had to: (a) guard a building with several entrances, (b) track a suspicious vehicle, and (c) scan the shoreline. All entrances and exits from and to the building were to be reported. When a suspect vehicle exited the building, the operator had to track it. Two UAVs were allocated to supervising the building entrances while one UAV was used for surveillance (lock-on target to track moving targets could be used). Each scenario was 4 min long and contained eight events that the participant had to attend to, events did not appear at the same time in the scene.

##### Results

Operators demonstrated difficulties in simultaneously processing information from three separate locations/video feed sources and failed to succeed in guarding the building and performing additional tasks such as tracking the moving vehicle or scanning the beach line. Only three operators out of the 10 were able to complete the scenarios at some degree of success the remaining seven had difficulties in performing the task and quit before the scenarios ended.

#### Study 2 summary

Experienced operators seemed to cope well with two video feed windows when using the Twin UAV setup. Interestingly, without being instructed to do so, operators intuitively enhanced their performance by utilizing the dual setup. One method that was used frequently by the operators was to choose a wide field-of-view (FOV) angle in one UAV for overview, and a narrow angle on the other UAV for recognition and tracking of the target. Furthermore, in this type of configuration, since the area of operation was limited, operators rarely used the map. In general, operators thought that handling two sources was difficult enough and that handling three devices may be too demanding. This proved itself correct in condition B, when operators had difficulties processing the information from the three video feed sources. Note also that the area of operation in condition B was wider. In order to succeed, operators stated that there was a need for automated supporting tools. Following these results, in study 3 an attempt was made to facilitate the task by providing the operators with a toolkit containing situation awareness enhancing indicators and decision-support tools.

Table 1 summarizes studies 1 and 2 as described above. For each study, cognitive task demand, and automation level was added in a separate column (in line with Cummings et al., 2007a). See Table 2 for the levels of automation legend.

**Table 1:** **Summary of studies 1 and 2**.

**Study/Condition**	**Description/Design**	**Operator/UV ratio**	Cognitive task and automation level^*^	**Results**
**1**	**UAVs HEALTH AND STATUS SUPERVISING**			
Initial state	Supervise 13 health indices using a paper-based checklist.	1:5	Health monitoring-I	Operators indicated that the task was difficult, exhaustive, and caused high workload.
A	Two design additions: 1. For each data item an intact indication was added 2. All UAV forms were displayed simultaneously.	1:5	Health monitoring -III	Improved performance from the Initial State.
A+	Display was the same as in condition A. However, five additional UAVs were added to the monitoring task.	1:10	Health monitoring -III	Similar results to condition A.
B	12 health indices were grouped into four meaningful groups. For each UAV, only the group indications were displayed on the form. For each group, three intact indications were displayed (intact, warning, and fault). The operator could open the full form by clicking on the indication group.	1:20	Health monitoring -III	Similar results to condition A. Operators reported high workload and a feeling of losing control after the 17th UAV was added.
B+	The group indications used in Condition B for each UAV were replaced with one intact indication (icon) placed on the command and control map. The operator could click on the icon and view the details. In addition, an alert was added for location deviation.	1:20	Health monitoring -III	Similar results to condition A, except for the time to detect deviation from route which was shortened. Operators succeeded in supervising 15–17 UAVs.
C	For each indicator, a graph displaying its measured values and intact indications was added.	1:20	Health monitoring-III	Similar results to condition A, except for the time to detect the fault source, which was shortened. Operators succeeded in supervising up to 10 UAVs.
**2**	**UAVs CONTROL—1-TO-1 AND 1-TO-MANY**			
A	Tracking a vehicle once with one UAV and once with a Twin UAV. Target lock-on could be used.	1:1 vs.1:2	Navigation—V	Performance was significantly better using two UAVs.
B	The operator had to guard a building with several entrances, track a suspected vehicle (target lock-on could be used), and scan the beach line. 2 UAVs were required for supervising the building while one UAV was required for surveillance.	1:3	Navigation—V Mission Management-II	Operators demonstrated difficulties in processing information from three separate sources.

* *See Table 2 for the levels of automation legend*.

**Table 2:** **Levels of Automation (LOA) (cf Cummings et al., 2007a)**.

SV^*^-LOA	C^**^−LOA	**Automation Description**
1	I	The computer offers no assistance; human must take all decisions and actions.
2	II	The computer offers a complete set of decision/action alternatives.
3	III	The computer offers a selection of decisions/actions.
4/5	IV	The computer suggests one alternative and executes that suggestion if the human approves (management by consent).
6	V	The computer suggests one alternative and allows the human a restricted time to veto before automatic execution (management by exception).
7/8/9/10	VI	The human is not involved in the decision making process; the computer decides and executes autonomously.

* *SV—The 10-level scale originally proposed by Sheridan and Verplank (1978)*.

** *C—The combined categories of Cummings et al. (2007a)*.

In the following studies performance of a team vs. a single operator was compared in an attempt to understand the feasibility and advantage of each mode, in the UAS domain (Study 3) and in the UGV (unmanned ground vehicle) domain (Study 4). Utilizing the DRM, and based on the findings of the previous studies, tools and visual aids were added to the interface, as specified in each study.

### Study 3

*Problem:* Identify advantages and disadvantages of an individual operator vs. a team. Performance of one operator was compared to a team of (2–4) operators controlling the same number of UAVs (up to four UAVs). Operators had to observe a building and report of vehicles entering and existing the building. Vehicles exiting the building that had specific characteristics had to be further processed.

Study Question: Will a team of operators controlling a number of UAVs perform better than one operator controlling the same number of UAVs?

#### Condition A—two operators vs. one

##### Task

2:2: vs. 1:2—Two operators sharing control of two UAVs compared to one operator controlling two UAVs.

##### Participants

Six highly experienced male operators (SMEs) with similar military background and skills participated in this condition. They had 2–7 years of experience in operating military UASs (mean: 4), and their age ranged from 23 to 27 (mean: 24.8).

##### Procedure

Operators had to observe a building and report of vehicles entering and exiting the building. Vehicles exiting the building that had specific characteristics (i.e., suspicious vehicle) had to be further processed (track and report). Two phases were conducted, in the first phase no additional unique interaction tools were provided. After the first phase, based on the findings from study 2 and the difficulties operators had in performing the task, supportive tools were provided, only to the single operator in a form of a toolkit. The toolkit consisted of spatial anchoring capabilities like “sketch” and “revisit,” which enabled the operator to request the system to automatically follow a pattern (perform a sketch) or a jump through a list of points (perform a revisit cycle) by generating (using mouse clicks) a list of points on top of the payload image. In a similar way to Study 2's Twin UAV setup “Payload coupling” enabled the operator to enslave one UAV to the other. Finally, “Camera guide” enabled the operator to fly the UAV by following its camera (See Oron-Gilad et al., 2011 for detailed description of several tools).

In phase 2 of the study, it was aimed to examine whether the toolkit could support the single operator's performance to a degree superior to the team of two operators.

##### Results

Results are displayed in Table 3.

**Table 3:** **Performance measures—Team of 2 vs. one operator controlling two UAVs**.

**Performance measure**	**Team of 2—2 UAVs**	**1 operator—2 UAVs**	**1 operator—2 UAVs + toolkit**
Misses of vehicle exits	2%	2%	3%
Misses of vehicle entrances	3%	4%	3%
Misses of a suspect vehicles	0.5%	0.7%	0.5%
Multiple reporting of the same vehicle	15%	5%	5%
Mission stabilization time	10 min (SD 2.5)	6 min (SD 1.75)	2 min (SD 0.8)

The team reported that the mission was calm up to a degree of being boring. The single operator reported that the mission was challenging but not overloading. The results of the team were similar to the results of the single operator using a toolkit. Multiple reporting of the same incident and longer mission stabilization time occurred in the team condition.

#### Condition B—three operators vs. one

##### Task

3:3 vs. 1:3—A team of three operators sharing control over three UAVs were compared to one operator controlling three UAVs. The same scenarios as in Condition A, the individual operator could use the toolkit and the operators in the team could not.

##### Participants

Eight highly experienced male operators (SMEs) with similar military background and skills participated in this condition. They had 4–8 years of experience in operating military UASs (mean: 5.4), and their age ranged from 25 to 30 (mean: 26.9). SMEs were compensated for their time.

##### Results

Results are displayed in Table 4.

**Table 4:** **Performance measures—Team of 3 vs. one operator controlling 3 UAVs**.

**Performance measure**	**Team of UAVs 3—3 UAVs**	**UAVs + toolkit 1 operator—3**
Misses of vehicle exits	1%	3%
Misses of vehicle entrances	1%	3%
Misses of a suspect vehicles	0%	0.5%
Multiple reporting of the same vehicle	25%	8%
Mission stabilization time	13 min (SD 3)	3.5 min (SD 1.2)

The team performed significantly better (*p* < 0.01) than the single operator, however they again had more occasions of multiple reporting of the same incident, and increased stabilization time.

#### Condition B+—four operators vs. one

##### Task

4:4 vs. 1:4—A team of four operators sharing control of four UAVs were compared to one operator controlling four UAVs.

##### Participants

Five highly experienced male operators (SMEs) with similar military background and skills participated in this condition. They had 3–5 years of experience in operating military UASs (mean: 3.83), and their age ranged from 25 to 27 (mean: 25.5).

##### Results

This setup was problematic to analyze. In the one operator condition, single operators felt lost looking at four video feeds and in some cases they just looked at three UAVs or less (hence they neglected the fourth UAV). In the team condition, coordination among the operators took a long time, containing incessant verbal communication, and numerous multiple reports.

#### Study 3 summary

One operator could not control more than three UASs, even with additional aids. Furthermore, without facilitating decision support tools, it was difficult and ineffective for a team of four operators to control four UASs as well. The implications of this study were twofold: each single operator can benefit from designated tools that assist in conducting the mission, e.g., coupling or sketch and revisit. A team of operators must be familiarized with a set of rules or provided with a set of tools to facilitate collaboration. Otherwise, they are prone to report multiple times on the same incident and they are not fully aware of each other's doings. Following these findings several novel tools and displays were designed to facilitate payload switching among members of the team (see for example Porat et al., 2011). Probably, the most successful facilitating tool was the “Castling Rays,” which is a switching decision aid, enabling operators to visually view which UAS has the best view of “their” target at any given moment (Porat et al., 2010).

### Study 4

*Problem:* There was a requirement to increase the number of UGVs that one operator can control. The main problem with UGVs is that their level of autonomy is lower, hence more attention needs to be allocated to navigation and driving issues than in UAVs. At the time tested, the problem domain was still within the realm of multiple operators controlling a single system vs. a single operator. We compared performance of two operators controlling (navigating and observing) one UGV to one operator controlling one UGV.

*Study Question:* Will two operators controlling one UGV perform better than one operator controlling one UGV for scanning the fence?

#### Initial state

##### Task

2:1—Two operators controlled one UGV: one operator performed the navigation task and one operator performed the observation task, while scanning a border fence.

##### Participants

Six highly experienced participants, reserve soldiers in an elite engineering unit with experience in controlling remote robots such as ANDROS and Mini-ANDROS participated in this condition. They had 2–6 years of experience in operating military UVGs (mean: 3.5), and their age ranged from 25 to 30 (mean: 26.8). All were compensated for their time.

##### Procedure

**Figure 7:**
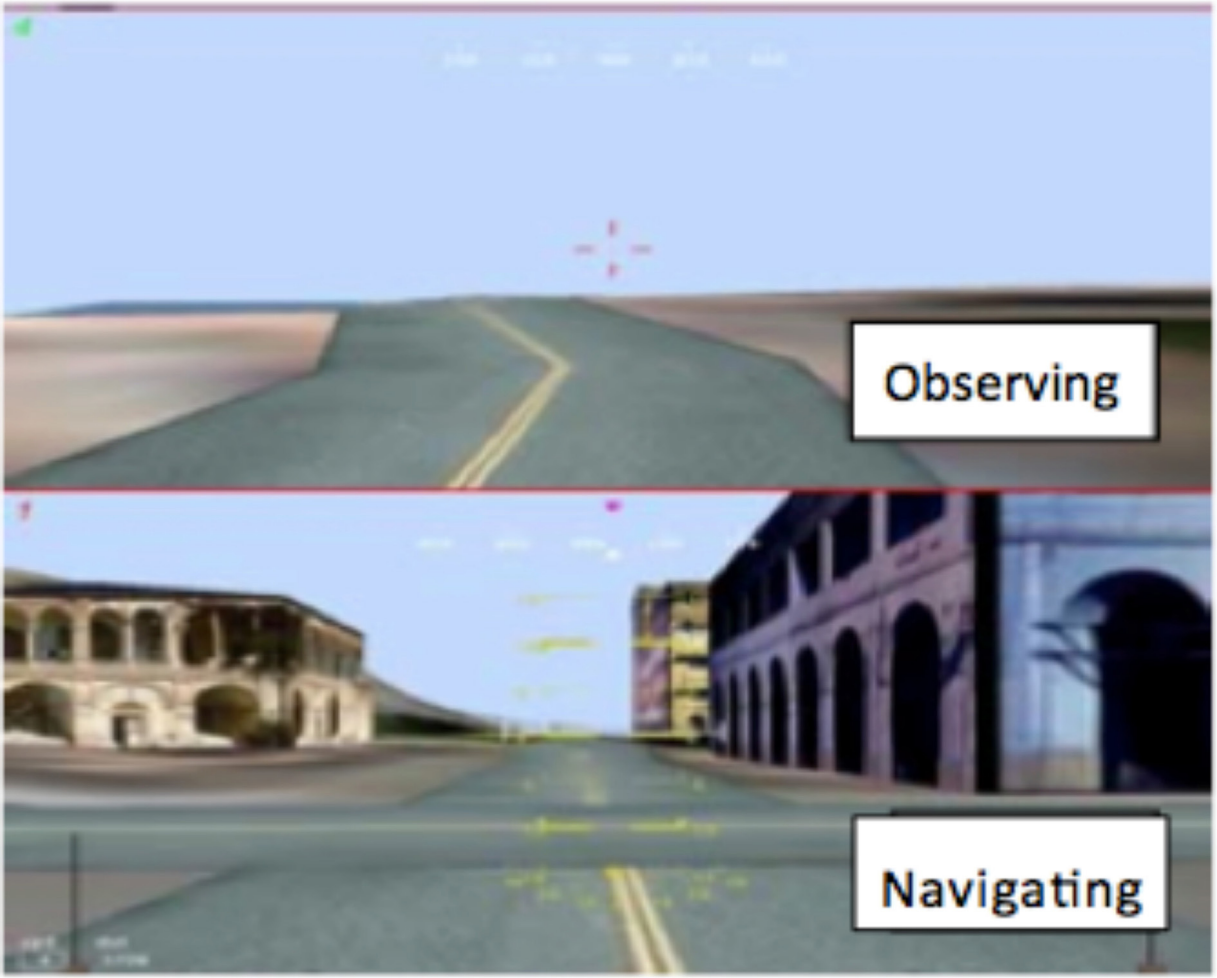
**Observing camera (above) and navigating camera (below)—Condition A**.

Each UGV had a navigation camera and an observation camera for scanning the fence for obstacles and hazards (Figure 7). The UGV moved very slowly (7 km/h). One operator performed the navigation task (including health monitoring—alerts were both color coded and audible), and one performed the observation task. The experimental trial took about an hour. In this period, a total of 100 events occurred (obstacle, hazard on the fence, fault in the vehicle).

##### Results

Results are displayed in Table 5.

**Table 5:** **Performance measures of the initial state**.

**Performance measure**	**Initial state**
Time to identify an obstacle	4 s (SD 1.5)
Missing an obstacle^*^	3%
Time to identify a hazard on the fence	3 s (SD 1.5)
Missing a hazard on the fence	2%

* *All missed obstacles were of type “pitfall.” Pitfalls are more difficult to identify than above ground hazards such as a log put on the ground*.

Performance was acceptable with a relatively low rate of misses of obstacles. However, there were synchronization problems among the two operators, for example: operators had delays in stopping the vehicle, which usually occurred after the observer identified a hazard, and notified the navigator who then had to stop the vehicle.

#### Condition A

##### Task

1:1—one operator controlled one UGV, performing both the navigation and the observation tasks (as shown in the display in Figure 7).

##### Participants

Three highly experienced participants, reserve soldiers in an elite engineering unit with experience in controlling remote robots such as ANDROS, and Mini-ANDROS participated in this condition. They had 2–4 years of experience in operating military UGVs (mean: 2.7), and their age ranged from 25 to 28 (mean: 26.3).

##### Results

Performance measures between the “Initial State” and “Condition A” were compared. Results are displayed in Table 6.

**Table 6:** **Performance measures—initial state vs. condition A**.

**Performance measures**	**Initial state**	**Condition A**
Time to identify an obstacle	4 s (SD 1.5)	7 s (SD 2.3)
Missing an obstacle^*^	3%	6%
Time to identify a hazard on the fence	3 s (SD 1.5)	9 s (SD 3)
Missing a hazard on the fence	2%	5%

* *All missed obstacles were of type “pitfall.” Pitfalls are more difficult to identify than above ground hazards such as a log put on the ground*.

One of the main problems in this condition was that operators were missing pitfalls, which stopped the vehicle and increased the time based performance measures to a large extent.

#### Condition B

##### Task

1:5—one operator **observed** cameras from five different UGVs, scanning the fence for obstacles and hazards.

##### Participants

The same participants as in condition A.

##### Results

Performance measures between the “Initial State” and “Condition B” were compared. Results are displayed in Table 7.

**Table 7:** **Performance measures—initial state vs. condition B**.

**Performance measures**	**Initial State**	**Condition B**
Time to identify an obstacle	4 s (SD 1.5)	5 s (SD 3)
Missing an obstacle^*^	3%	4%
Time to identify a hazard on the fence	3 s (SD 1.5)	4.5 s (SD 2.5)
Missing a hazard on the fence	2%	3%

* *All missed obstacles were of type “pitfall.” Pitfalls are more difficult to identify than above ground hazards such as a log put on the ground*.

#### Study 4 summary

It was too complicated for one operator to perform the observation and navigation tasks simultaneously (as in Condition A). These two task types require different skills and performing them at the same time generated major switching costs. However, when operators were performing only one type of task (observation or navigation), their performance has improved.

**Figure 8:**
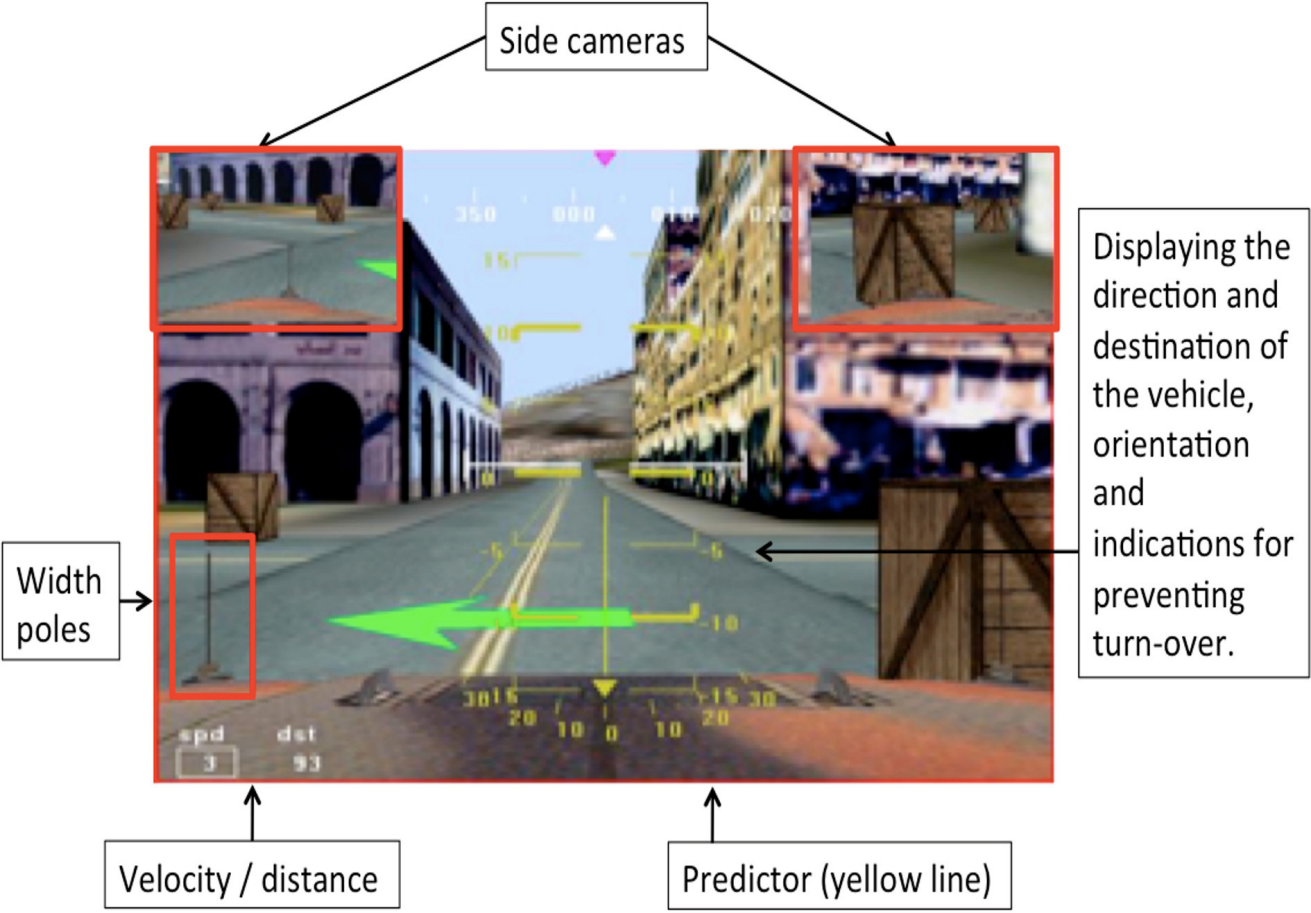
**Display of the navigation cameras with additional supporting tools and displays**.

Based on these findings, several novel tools and displays were designed to facilitate the navigation task, as shown in Figure 8. Side cameras were added. A width pole display aided the operator in estimating the width of the vehicle, and a path Predictor displayed a virtual path that the navigator could follow. Initial examination found this setup to decrease navigation time and improve navigation accuracy. This needs to be further assessed, however could be extremely useful especially when there are communication delays in displaying the online video feed from the navigation cameras.

Table 8 summarizes studies 3 and 4 as described above. For each study, cognitive task demand and automation level were added in a separate column (in line with Cummings et al., 2007a). See Table 2 for the levels of automation legend.

**Table 8:** **Summary of studies 3 and 4 (MOMU)**.

**Study/Condition**	**Description/Design**	**Operator/UV ratio**	Cognitive task and automation level^*^	**Results**
**3**	**UAVs CONTROL—1-TO-MANY VS. MANY-TO-MANY**			
A	The operator had to observe a building and report of vehicles entering and exiting the building. Vehicles exiting the building that had specific characteristics had to be further processed. The single operator could use a toolkit containing decision-making tools.	1:2 vs. 2:2	Team: Mission Management- I Single: Mission Management-I + Tools	Teams of two operators described the mission as calm (and even boring). The single operators reported that the mission was challenging but not overloading.
B	The same scenarios as in Condition A. The single operator could use a toolkit and the team of operators could not.	1:3 vs.3:3	Team: Mission Management-I Single: Mission Management-I + Tools	The team performed better than the single operator. However, the team had more double reporting.
B+	The same scenarios as in Condition A. The single operator could use a toolkit and the team of operators could not.	1:4 vs. 4:4	Team: Mission Management-I Single: Mission Management-I + Tools	This setup could not be examined since the tasks were too complex.
**4**	**UGVs CONTROL—MANY-TO-1 AND 1-TO-1**			
Initial	One operator performed the navigation task and one performed the observation task.	2:1	Navigator: Navigation-I Observer: Mission Management-I	Performance was satisfactory. Synchronization problems between the two operators were apparent.
A	One operator performed the navigation and the observation tasks.	1:1	Navigation-I Mission Management-I	It was too complicated for one operator to perform both observation and navigation tasks.
B	One operator observed five UGVs.	1:5	Mission Management-I	Performance was good and similar to the initial condition.

* *See Table 2 for the levels of automation legend*.

## Summary and discussion

In general, our results suggest that one experienced operator can supervise (system health and status) up to 15 UASs efficiently using moderate levels of flight control automation. Concerning controlling UASs (mission and payload management), one experienced operator cannot control more than three UASs, with the level of complexity and automation that has been examined. Providing the operator with various display aids and decision support tools does improve performances of a single operator (as in Study 3) but did not change the modal number to higher extents.

Automation level, availability of decision aids, operators' experience, complexity and criticality of the mission, operational tempo, and cognitive resources and demands, all influence the number of systems that one operator can control. For this reason, comparison across studies is often complicated and inaccurate. However, considering these limitations, our findings do resemble findings of previous studies in the essence that they are confirming that single operators are able to control more remote vehicles as they are provided with increasing automated decision support. Given some automated navigation assistance and management-by-consent automation in the mission management loop, an operator was able to control 4–5 vehicles (e.g., Ruff et al., 2004; Dunlap, 2006; Cummings et al., 2007b). A leap in the amount of vehicles that one operator could control was only seen if management-by-exception was introduced, increasing the number to 8–12 vehicles (e.g., Lewis et al., 2006; Cummings and Guerlain, 2007). Here, we were able to show via Study 1 that a single operator can achieve even a higher ratio of operation between 15 and 17 systems, but only on a limited task or mission component (e.g., health monitoring).

This finding may become more relevant in the future, if organizations change the way they allocate and recruit operators. Nowadays, most organizations, military amongst them, do not want to parse their operators' mission into “small” subtasks and create high levels of skills in fine grained subtasks of the mission among operators (i.e., train people to be experts only on a single component of the mission, such as taxi or health supervising). The current approach can be justified when considering the danger of having operators lacking skill while conducting dynamic, time critical, and situation critical missions. However, the way operators' allocations are done today, it is inevitable for operators to maintain a certain level of proficiency in all aspects of their mission. Evidently this setting dictates that the level of automation of the unmanned system and the use of decision aids become key considerations.

**Figure 9:**
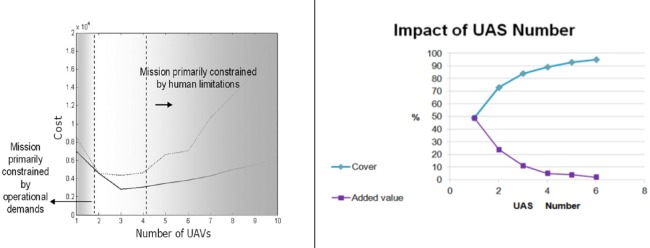
**Left—operator capacity as a function of mission constrains (cf Cummings et al., 2007b)**. Right—Impact of UAS Number from an OR study conducted in parallel to our studies (Shaferman and Shima, 2009).

Human operators are vital in this critical, high risk and high demand environment. Keeping the human in the loop, mostly for planning, re-planning, and control or at least for being able to take over in automation malfunction is essential in this domain. Therefore, fully autonomous operations (automation level VI) are not expected any time soon. Using intermediate levels of automation (i.e., supervisory control), will not enable operators to exceed the control of few systems. Figure 9 on the left was taken from Cummings et al. (2007b) and shows that the optimal bound they found was between 2 and 4 vehicles. The left region is primarily constrained by operational demands, but the right region is dominated by human performance limitations. Figure 9 on the right is taken from an operation research study conducted in parallel to Studies 1–4 and on similar urban area conditions (Shaferman and Shima, 2009). It shows that adding the first and second UAV had the most significant influence on mission performance. Above four systems, the area covered, and the added value of more assets became negligible. Hence, organizations need to identify whether there are justified operational cases where one-to-many ratios of more than four are needed. If those cases are sparse, then perhaps more design effort can be geared toward sharing of assets among operators (MOMU) in an efficient and effective way.

Concerning the operation of UGVs, when the operator performed only one task, as in study 4, condition B (observation task), performance was satisfactory since the operator focused primarily on maintaining awareness for obstacles and hazards. However, when the operator had to navigate the vehicle and observe the fence (as in study 4, condition A), it was too complicated to perform. Dynamic task switching between different functions resulted in greater cognitive workload for the operator than performing only one type of task. In both UASs and UGVs, the human and the automated systems are geographically separated, and therefore face difficulties, which are inherent in remote perception, such as overcoming the “keyhole” or “soda straw effect” (Voshell et al., 2005). Controlling and navigating UGVs is more complex than UASs with regard to spatial perception. While GPS technology may be very effective in providing UASs with positioning information that meets their navigational needs, their use in UGVs may be limited by reliability and accuracy constraints (Chaimowicz et al., 2005). For example, a positioning error of one or two meters may have little effect when controlling a UAS, however it could have crucial results when navigating a UGV.

Successful interaction with any human and automated system is influenced by many factors including vehicle characteristics (air, ground), task characteristics (complexity, number of vehicles controlled, time pressure, workload), environmental characteristics (terrain characteristics, quality, obstacles), and technological constraints (available bandwidth, communication delays). Thus, design specifications of automated decision support aids will differ according to the unique needs of the human operator in each situation. Indeed, the decision support tools that were developed in this study for the aerial and the ground domain differ in their design and implementation (e.g., width pole display for the ground vehicle) but there are also many commonalities in the essence of things (e.g., coupling of vehicles is suitable for both aerial and ground vehicles).

In MOMU environments, as seen in Study 3, when the tasks are similar or when the interest areas overlap (i.e., a connection between the video feeds), one operator has an advantage to a team who need to collaborate and coordinate. However, when there is no connection between the video feeds, a team has an advantage to a single operator. Thus, one of the considerations to prefer one operator to a team is the amount of overlap between the different video sources covered by the payloads. Taking this findings to a practical level, in the MOMU operational settings we strive to gain a consistent ratio of one operator controlling two UASs with some flexibility, thus controlling up to three UASs per operator on demand, and supervise up to six UASs where the covered areas of the UASs are related.

## Where can we go from here and broaden the understanding and added value of MOMU environments?

The first notion is that automation is a tricky tool. When not tailored to the task, it can easily cause high operator workload, and challenge the “keep the human in the loop” principle. Although this statement may seem true for most human system interfaces, when applying automation in critical and complex environments, such as MOMU, a first step would be to perform a thorough behavioral and cognitive task analysis to understand the cognitive requirements of the task (e.g., decisions, situation awareness, cues, judgment points). Once the different tasks, requirements and possible errors are understood, tailoring the display design and the automation level to the desired setup becomes possible. It should be acknowledged that different sections/parts/sub-tasks of the entire mission are perceived differently at separate stages of the mission process. For example, different automation needs are required for locomotion between areas of interest, as opposed to loitering on a specific target area. This implies that Cummings et al. (2007a) control loops could be further divided into even smaller chunks, and for each chunk one should match the required and desired automation level.

The second notion is that the scenarios used in our studies assumed similarity: all operators had the same type of experience and training, and all systems were alike. While this is a typical mode of operation, it is evident that this is just one possibility. In the U.S. military operations in Iraq, for example, more than 100 UASs of 10 different types were used (Office of the Under Secretary of Defense for Acquisition, 2004). The rising question becomes how MOMU operations may vary if there were multiple types of vehicles and operators with various training and capabilities. One needs also to reconsider the traditional mission allocation. Recent studies tried to define the qualifications and training required from an operator that is expected to control an increasing number of UASs. Parasuraman et al. (2014) discussed the possibility of selecting and training operators according to their molecular genetics. Perhaps now is the time to initiate specialization of operator roles. In order to do so, it would be necessary to revisit the main operational tasks and reallocate them in view of mission benefit. Changes in the function allocation and the nature of task differentiation between human operators and unmanned systems could significantly alter the cognitive loads of the operators when performing the mission (Cuevas et al., 2007). We should introduce flexibility into our rigid-traditional “task thinking,” and let go of beliefs that tie us down and stop the evolvement: must human operators fly the platform? Can we mentally not technically—enslave the platform to the mission needs?

A third direction would be to develop tools and decision-making aids. In our studies tools and techniques that may facilitate operators in MOMU environments were introduced (e.g., Porat et al., 2010). Tool development was done in a bottom up approach, i.e., based upon needs retrieved from SMEs and geared toward solving particular challenging operational situations. Since the tools were not yet tested in real world settings, it would be interesting to examine how they integrate into UASs MOMU environments and affect the metrics of performance. Fern et al. (2011) for example proposed other alternatives to facilitate UAS MOMU operations. It would also be interesting to examine whether tools can be transferred to other MOMU settings such as ground vehicles or drones.

Fourth, our studies focused on the allocation among operators in one team while conducting a single scenario, one can start looking at the broader picture—how to break operations into teams, how to assign and allocate the correct number of assets and operators to each one of the teams, and how to coordinate among teams of MOMU operators.

All these former suggestions lead toward the notion that a more top-down approach needs to be developed in order to provide a coherent way to distribute responsibilities and tasks in MOMU environments. This direction of adjusting resources and personnel according to mission needs is in line with future intentions and models in other domains. For example, in the medical domain, the NHS recent report “Five year forward view” (NHS England, 2014), argues that England is too diverse for a “one size fits all” care model, services need to be integrated around the patient and support their changing needs. Different local health communities will be able to decide which care delivery model best supports their needs, such as a multispecialty community provider model which is a multi-disciplinary team that can include different specialties such as nurses, therapists and other professionals combined with the latest digital technologies, or a specialized care model which is a surgery that specializes in one area such as cancer and provides care only for these patients. All to support the main goal, which is providing the best care for patients. Translating this to our domain and the task specific requirements, we can reach to the extreme cases where a team of operators will control only one asset and vice versa, where a single operator will control up to 15 assets simultaneously (e.g., taxi).

Finally, with regard to Human-Robot Interaction, it is inevitable that people of various abilities and skills will be surrounded by multiple platforms of various kinds and autonomy levels. Much of what is now known from the realm of UASs can be used to facilitate efficient asset sharing and mission successes among other populations. Just to mention one, in the not so far future, the elderly community will be utilizing robotics assistants of various kinds, whether operated by caregivers or by the users themselves. Many of the questions that were raised here about operators' skills, tools to facilitate cooperation and sharing and mission accomplishment will be relevant to these domains as well.

## Author contributions

Conceived and designed the experiments: MR, JS. Analyzed and interpreted the data: TP, TO, MR, JS. Wrote the paper: TP, TO, MR.

### Conflict of interest statement

The authors declare that the research was conducted in the absence of any commercial or financial relationships that could be construed as a potential conflict of interest.

## Footnotes

^1^*Office of the Secretary of Defense, “Unmanned Aircraft Systems (UAS) Roadmap, 2005-2030.”* Washington DC: DoD.

^2^In 1954, Taylor and Birmingham, published a paper in the Journal of the Institute of Radio Engineers (now IEEE), titled “A Design Philosophy for Man-Machine Control Systems” (Birmingham and Taylor, 1954). The article discussed the manual control of a submarine, which is a complex control problem because of the massiveness of the boat and the nature of the control surfaces. They also described “quickening”, a clever example of how one could augment the display of information to improve the stability of control.
